# Italian multidisciplinary delphi consensus on home management of nausea and vomiting in children with acute gastroenteritis (NoEmesi Project)

**DOI:** 10.3389/fped.2026.1773341

**Published:** 2026-04-14

**Authors:** Ruggiero Francavilla, Fernanda Cristofori, Gian Luigi Marseglia, Antonio D’Avino, Giorgio Ciprandi, Michele Miraglia del Giudice

**Affiliations:** 1 Interdisciplinary Department of Medicine, Pediatric Section, Children’s Hospital ‘Giovanni XXIII’, University of Bari Aldo Moro, Bari, Italy; 2 Department of Clinical, Surgical, Diagnostic and Pediatric Sciences, University of Pavia, Pavia, Italy; 3 Pediatric Clinic, Fondazione IRCCS Policlinico San Matteo, Pavia, Italy; 4 Italian Federation of Pediatricians (FIMP), Naples, Italy; 5 Allergy Clinic, Casa di Cura Villa Montallegro, Genoa, Italy; 6 Department of Woman, Child and General and Specialized Surgery, University of Campania “Luigi Vanvitelli,” Naples, Italy

**Keywords:** acute gastroenteritis, antiemetics, child, delphi consensus, metoclopramide, nausea, vomiting

## Abstract

**Introduction:**

Vomiting in pediatric acute gastroenteritis (AGE) is a major driver of distress, caregiver anxiety, and failure of oral rehydration, while guidance for home management and pediatric antiemetic use is limited and influenced by regulatory restrictions.

**Methods:**

We conducted a modified Delphi consensus in three phases (statement development after literature review, anonymous online rating, and final refinement) involving a national multidisciplinary panel of 70 Italian pediatricians.

**Results:**

Twenty-one statements addressing clinical aspects of vomiting in AGE and the pharmacology, safety, and use of commonly prescribed antiemetics (with emphasis on metoclopramide) were rated on a five-point Likert scale; consensus was predefined as ≥80% ratings of 4–5. All 21 statements met the consensus threshold (agreement 83%–100%; mean scores 4.2–4.9). The highest agreement concerned the clinical burden of vomiting in AGE, key mechanistic pathways, and practical safety considerations for metoclopramide (including dosing limits, age-related caution, and caregiver counseling). The panel agreed that, in selected children with persistent vomiting preventing oral rehydration, cautious antiemetic use, particularly metoclopramide, may support home management when safety precautions are followed, off-label requirements are respected, and parents are adequately counseled.

**Discussion:**

This Delphi consensus provides pragmatic, expert-endorsed recommendations for home management of nausea and vomiting in pediatric AGE. Oral rehydration solutions (ORS) remain the cornerstone of care; metoclopramide may be considered in carefully selected cases to quickly resolve symptoms, facilitate rehydration and reduce dehydration risk, provided that clinicians adopt strict risk–benefit assessment and good clinical practice safeguards.

## 1 Introduction

Acute gastroenteritis (AGE) is one of the most common medical conditions in childhood and represents a major source of discomfort for children, concern for caregivers, and healthcare utilization worldwide (1–4). Beyond its immediate clinical impact, AGE is associated with substantial morbidity, including school absenteeism and loss of productivity for families, and contributes significantly to healthcare costs (2, 3). In more severe cases, AGE may lead to dehydration and electrolyte disturbances requiring intravenous rehydration and hospitalization, thereby amplifying its socioeconomic burden.

AGE is characterized by acute inflammation of the gastrointestinal mucosa and is most frequently caused by viral pathogens, including rotavirus, norovirus, adenovirus, and astrovirus (3). Among bacterial etiologies, Salmonella, Shigella, Campylobacter, and Escherichia coli are the most commonly implicated agents (4). Transmission typically occurs through ingestion of contaminated material, most often via the fecal–oral route.

Although diarrhea is often considered the hallmark symptom of AGE and dominates most guideline frameworks, vomiting plays a pivotal role in real-world clinical management. Vomiting directly interferes with the effectiveness of oral rehydration therapy (ORT), increases distress in affected children, heightens parental anxiety, and is a key driver of emergency department referral. Persistent or severe vomiting may precipitate dehydration and electrolyte imbalance, frequently prompting escalation of care and hospital admission (3).

In Italy, the majority of pediatric AGE cases are managed at home by primary care pediatricians, with referral to hospital services reserved for selected situations. Standard management is centered on ORT and careful assessment of hydration status, complemented by symptomatic treatments. Current European evidence-based guidelines issued by the European Society for Pediatric Gastroenterology, Hepatology, and Nutrition (ESPGHAN) emphasize the central role of diarrhea and dehydration assessment in AGE management (5). While vomiting is acknowledged as a relevant symptom, it is primarily discussed in relation to its impact on dehydration and the feasibility of ORT. Accordingly, these guidelines dedicate only limited space to antiemetic therapy, mainly as a potential adjunct to facilitate rehydration (5).

Despite this relatively limited emphasis in guidelines, antiemetics, most commonly metoclopramide, domperidone, and ondansetron, continue to be widely used in both outpatient and inpatient pediatric settings. Their efficacy and safety have been evaluated in multiple clinical trials, systematic reviews, and meta-analyses, yielding heterogeneous results (6–23). Nonetheless, the role of antiemetics in pediatric AGE remains debated, and significant variability exists in prescribing practices and clinician attitudes, with some pediatricians remaining reluctant to use these agents in children (24, 25). A recent position paper addressing priorities in childhood AGE management highlighted the utility of ondansetron in emergency department settings, particularly to reduce the need for intravenous rehydration and hospital admission (26); however, it did not provide guidance for home management.

In parallel, regulatory restrictions and safety warnings issued in recent years have progressively narrowed the approved indications for several antiemetics in pediatric patients, creating a discrepancy between regulatory positions, available clinical evidence, and long-standing real-world practice. These limitations do not fully reflect the complexity of clinical decision-making in primary care, where antiemetics have been used for decades as part of the therapeutic approach to persistent vomiting. As a result, the optimal and safe use of antiemetic therapy in the home management of pediatric AGE remains controversial.

Given this gap between clinical practice, evidence, and formal guidance, there is a clear need for practical, safety-oriented recommendations tailored to the outpatient setting. To address this unmet need, we conducted a national, multidisciplinary Delphi consensus initiative (NoEmesi Project) aimed at developing expert-based recommendations for the home management of nausea and vomiting in children with AGE, with particular focus on appropriate indications, safety considerations, and rational use of antiemetic agents.

## 2 Materials and methods

### 2.1 Study design

This multidisciplinary consensus was developed using a modified Delphi methodology, in accordance with contemporary methodological recommendations for healthcare consensus processes (27). The Delphi approach was chosen to systematically integrate expert opinion in an area characterized by heterogeneous evidence, regulatory constraints, and variability in clinical practice.

The process consisted of three sequential phases: (i) a preparatory phase including literature review and statement development by a steering committee (Round 1); (ii) anonymous online rating of statements by a multidisciplinary expert panel (Round 2); and (iii) structured feedback, discussion, and final refinement of the consensus document based on aggregated results (Round 3).

### 2.2 Steering committee and preparatory phase (round 1)

A steering committee (Experts’ Board) was established, composed of clinicians with recognized expertise in pediatric gastroenterology, infectious diseases, primary care pediatrics, hospital pediatrics, and clinical pharmacology. Selection was based on long-standing clinical experience and scientific authorship in peer-reviewed, high-impact journals. The steering committee conducted a comprehensive literature review on the management of pediatric AGE, with particular focus on nausea and vomiting, antiemetic therapy, safety profiles, and regulatory recommendations. Specific attention was dedicated to the available evidence on metoclopramide use in children.

Based on the literature review, the committee drafted an initial set of 21 statements addressing: (a) the clinical relevance and burden of vomiting in AGE; (b) the pathophysiological mechanisms underlying nausea and vomiting; and (c) pharmacological characteristics, safety issues, and practical use considerations of commonly prescribed antiemetic agents.

Statements underwent internal revision until full agreement was reached within the steering committee, after which they were advanced to the panel evaluation phase.

### 2.3 Panel selection and composition

The steering committee subsequently invited a multidisciplinary panel of Italian clinicians actively involved in the management of pediatric AGE, aiming to ensure both professional and geographic heterogeneity. Selection criteria included recognized clinical expertise, scientific activity, and leadership within the respective specialty.

The final panel consisted of 70 participants, including academic pediatricians (*n* = 10), primary care pediatricians (*n* = 30), hospital pediatricians (*n* = 9), pediatric gastroenterologists (*n* = 5), pediatric surgeons (*n* = 3), pediatric oncologists (*n* = 3), and physicians in pediatric specialty training (*n* = 10). Panelists were geographically distributed across Italy to enhance representativeness.

### 2.4 Educational phase and delphi rounds (rounds 2 and 3)

Before the formal rating of statements (Round 2), all 70 panelists participated in a formative webinar designed to ensure a common baseline understanding of the key clinical issues, available evidence, and regulatory context relevant to vomiting in pediatric AGE.

Webinar content and delivery: The webinar was delivered by two members of the Steering Committee (R.F. and F.C.), both academic pediatricians affiliated with the University of Bari Aldo Moro, with no conflicts of interest related to the pharmaceutical industry. The educational content was standardized using a predefined slide deck developed collectively by the entire Steering Committee. The presentation provided a balanced overview of: a) the epidemiology and clinical burden of vomiting in pediatric AGE; b) current international guidelines (ESPGHAN) and their limitations regarding home management and antiemetic use; c) the pathophysiology of nausea and vomiting, including neurotransmitter pathways; d) a comprehensive and impartial review of the efficacy and safety profiles of commonly used antiemetics (metoclopramide, domperidone, ondansetron), including: findings from clinical trials, systematic reviews, and meta-analyses (both positive and negative); e) regulatory restrictions and safety warnings issued by the European Medicines Agency (EMA) and the Italian Medicines Agency (AIFA); f) conflicting evidence and areas of ongoing debate; g) non-pharmacological options (e.g., ginger), with explicit discussion of the limited evidence supporting their use.

The presentation was designed to present conflicting evidence and safety concerns in a balanced manner, ensuring that panelists were exposed to the full spectrum of scientific opinions before voting. No pharmaceutical company or commercial entity had any role in developing or delivering the educational content. Logistical support for the webinar platform was provided by Biomedia (Milan, Italy), an agency with no input into the scientific content.

Delphi rounds: Following the webinar, in Round 2, all statements were rated individually and anonymously using a secure, web-based platform. Aggregated results from Round 2 were subsequently presented during a structured feedback session (Round 3), allowing open discussion of areas of lower agreement in accordance with Delphi best practices. Throughout the process, individual responses remained anonymous and were not identifiable by other participants or the steering committee.

### 2.5 Rating scale and consensus definition

Each statement was rated using a five-point Likert scale: 1 = strongly disagree; 2 = disagree; 3 = no opinion; 4 = agree; 5 = strongly agree. Responses were categorized as negative (scores 1–2), neutral (score 3), or positive (scores 4–5). For each statement, the mean Likert score and the percentage of positive ratings were calculated. Consensus was defined *a priori* as at least 80% of panelists assigning a score of 4 or 5, in line with internationally accepted standards for Delphi consensus in healthcare research. For each statement, the percentage of positive agreement (scores 4–5), mean Likert score, median score, and interquartile range (IQR) were calculated. Given the ordinal nature of Likert-scale data, median and IQR were included to provide a robust measure of central tendency and dispersion. Response rates and completeness of data were recorded for each Delphi round.

### 2.6 Finalization of the consensus document

After completion of Rounds 2 and 3, the steering committee reviewed the quantitative voting results and qualitative comments. As all statements reached the predefined consensus threshold, no additional voting rounds were required.

The Experts’ Board therefore proceeded with drafting the final consensus document. The complete set of statements, along with detailed voting results, is reported in Table 1.

**Table 1:** Statements included in the multidisciplinary delphi consensus on the management of children with acute gastroenteritis (NoEmesi project) and relevant answers.

Statement	% agreement (scores 4 + 5)	Mean score (SD)	IQR
1. Acute gastroenteritis is a fairly common illness that mainly affects children. Acute gastroenteritis is a condition involving inflammation of the lining of the stomach and intestines. The main symptoms of acute gastroenteritis are vomiting, nausea, diarrhea, dehydration and abdominal pain, with possible fever and headache	100%	4.8 (0.2)	0
2. Vomiting is an extremely common symptom in children and is usually preceded and accompanied by a feeling of nausea. Vomiting causes distress in children and apprehension in parents. If uncontrolled, vomiting can lead to dehydration and electrolyte imbalance, making rehydration an important measure, but vomiting itself can counteract this. Vomiting can have many causes, but the most common in children is acute gastroenteritis	98%	4.76 (0.3)	0
3. Acute gastroenteritis is one of the most common causes of hospitalization in children. Therefore, careful management of nausea and vomiting at home and prevention of dehydration are measures that can have a significant socio-economic impact	98%	4.6 (0.4)	0.5
4. Acute gastroenteritis is usually a self-limiting condition that can be treated with oral rehydration at home. However, persistent and severe vomiting is unpleasant and stressful, and increases the likelihood of dehydration and electrolyte imbalances. In some cases, intravenous rehydration in hospital is necessary	98%	4.8 (0.1)	0
5. In children with uncontrollable vomiting who cannot be rehydrated orally at home, drug therapy may be used to promote oral rehydration and prevent admission to the emergency department. There are several drugs with antiemetic properties; the most commonly used are metoclopramide, domperidone and ondansetron. However, it is essential to understand their mechanism of action and safety profile	86%	4.2 (0.5)	1
6. Nausea and vomiting are coordinated by a complex neuroanatomical network involving peripheral vagal afferents, the area postrema, the nucleus tractus solitarius, and central pattern generators within the brainstem. Gastrointestinal inflammation activates vagal afferent fibers projecting to the nucleus tractus solitarius, while circulating emetogenic stimuli are detected by the area postrema, a circumventricular structure lacking a complete blood–brain barrier. These interconnected structures integrate dopaminergic, serotonergic, histaminergic, and cholinergic receptor-mediated signaling pathways, ultimately generating the motor and autonomic components of the emetic reflex. Pharmacological antagonism of dopamine and serotonin receptors modulates these pathways and may reduce vomiting in acute gastroenteritis	96%	4.6 (0.3)	1
7. Metoclopramide is a drug used primarily as an antiemetic and gastroprokinetic agent, which acts by essentially blocking dopamine receptors, but also partially blocking serotonin receptors in the gut and brain	100%	4.9 (0.1)	0.5
8. Metoclopramide blocks dopamine D2 receptors in the area postrema of the brain (chemoreceptor trigger zone), effectively reducing nausea and vomiting. In the gastrointestinal tract, the same D2 antagonism removes the inhibitory effects of dopamine on motility, increasing gastric contractility and facilitating emptying. Metoclopramide also has agonist activity on 5-HT4 receptors, further stimulating intestinal motility, and antagonist activity on 5-HT3 receptors, contributing to the antiemetic effect.	100%	4.8 (0.1)	0.5
9 The most common side effects include drowsiness, restlessness and diarrhea. Less frequent symptoms are extrapyramidal symptoms, which are usually reversible upon discontinuation	92%	4.6 (0.6)	1
10. The liquid formulation in drops (no longer commercially available) was previously the most frequent cause of overdose and, consequently, of adverse events. The use of the suspension formulation (syrup), which can be administered using dedicated measuring devices, reduces the risk of overdose and therefore the risk of adverse events	90%	4.4 (0.6)	1
11. Metoclopramide has good oral bioavailability even in children. Among the risk factors identified for the occurrence of adverse events, younger age (particularly under 5 years), body weight (which influences drug exposure), and female sex (especially in adults) should always be considered. The higher risk in very young children is related to age-dependent pharmacokinetic and pharmacodynamic factors,	96%	4.7 (0.2)	1
12. Caregivers of children receiving antiemetic therapy should be explicitly instructed to recognize early signs of adverse neurological reactions, including abnormal movements, muscle rigidity, oculogyric crisis, or acute dystonia. In the event of such symptoms, the medication must be immediately discontinued and urgent medical evaluation sought. Caregivers should not attempt home treatment of suspected extrapyramidal reactions	96%	4.7 (0.1)	0
13. The standard pediatric dosage (ages 1–18 years) of metoclopramide is 0.1–0.15 mg/kg up to three times a day, for a maximum of 5 days. However, the maximum dose in 24 h should not exceed 0.5 mg/kg of body weight. Regulatory agencies recommend careful use in children under 5 years of age	100%	4.9 (0.1)	0.5
14. Metoclopramide is available in three formulations: syrup, tablets and vials for parenteral use (in 2014, drops were withdrawn from the market). It allows for considerable adaptability of use	94%	4.3 (0.6)	1
15. Domperidone is only indicated for individuals aged 12 years and older and weighing at least 35 kg. Among the adverse events described is the possible prolongation of the QT interval, which may predispose to ventricular cardiac arrhythmias	100%	4.8 (0.1)	0,5
16. Ondansetron is indicated for chemotherapy-induced and post-operative nausea and vomiting in children. Although not formally approved for acute gastroenteritis (AGE), systematic reviews and meta-analyses, including a Cochrane review 22, demonstrate its effectiveness in reducing vomiting episodes and the need for intravenous rehydration in emergency department settings. However, robust evidence for its use in home management is lacking. Potential risks include QT prolongation, particularly relevant in children with electrolyte imbalances (hypokalemia, hypomagnesemia) due to fluid losses, and possible worsening of diarrhea. Rare cases of serotonin syndrome have been reported	83%	4.2 (0.5)	1
17. Among the non-pharmacological remedies proposed for the home management of vomiting in children, ginger (standardized ginger extracts) represents a potential symptomatic approach. However, the available evidence of its efficacy is currently very limited	93%	4.4 (0.3)	1
18. Early home treatment of vomiting with medication can prevent severe dehydration, facilitate oral rehydration, reduce the need for parenteral rehydration and thus prevent visits to the emergency room	86%	4.2 (0.4)	1
19. Quick relief from nausea and vomiting reduces distress for the child and anxiety for parents	100%	4.9 (0.1)	0
20. There is evidence in the literature that metoclopramide, even when administered orally, effectively controls vomiting	92%	4.4 (0.1)	1
21. In children with acute gastroenteritis and uncontrollable vomiting that does not respond to standard measures, the use of metoclopramide may be considered under medical supervision, with careful assessment of the risk-benefit ratio and prior information to parents	88%	4.3 (0.6)	1

### 2.7 Ethical considerations

This study involved the collection of opinions from a panel of expert clinicians through a structured Delphi consensus process. The study did not involve patients, medical interventions, or the collection of sensitive personal data.

Ethics review and approval: In accordance with Italian national regulations [including the Italian Medicines Agency (AIFA) guidelines on non-interventional studies and Legislative Decree 211/2003, which pertains to clinical trials and does not apply to this type of consensus research involving only healthcare professionals], formal approval from an Institutional Review Board (IRB) or Ethics Committee was not required. This waiver was confirmed by the coordinating center (University of Bari Aldo Moro). The study protocol was reviewed by the Steering Committee to ensure adherence to ethical principles and research integrity standards.

Informed consent: All 70 participating panelists were fully informed about the scope of the project, the voluntary nature of their participation, and the intended use of aggregated data for research and publication purposes. This information was provided in the invitation email and reiterated at the beginning of the web-based survey. Participation in the study (i.e., completing the anonymous online survey in Round 2) was considered to imply informed consent.

Anonymity and data protection: To ensure anonymity and confidentiality, the online rating platform was configured to prevent the collection of IP addresses or any personally identifiable information linked to individual responses. Each panelist received a unique, non-identifiable link to access the survey. All data were aggregated for analysis, and no individual responses are identifiable in this manuscript. Data were stored on a secure, password-protected server compliant with the EU General Data Protection Regulation (GDPR) 2016/679, accessible only to the principal investigators.

All procedures were conducted in accordance with the ethical standards of the Declaration of Helsinki and applicable data protection regulations.

### 2.8 Funding

This consensus initiative did not receive any financial support from pharmaceutical companies, commercial entities, or external sponsors. No educational grants or industry funding were provided for the development of this project. Biomedia (Milan, Italy) provided logistical and technical support for the organization of the educational webinar and the management of the online Delphi platform. Importantly, Biomedia had no role in the study design, the development of scientific content, the literature review, the formulation of statements, the analysis or interpretation of data, the writing of the manuscript, or the decision to submit it for publication. All scientific decisions, including the content of the educational materials and the final consensus statements, were made independently by the Steering Committee. The authors maintained full scientific independence throughout the entire project.

## 3 Results

**Figure 1:**
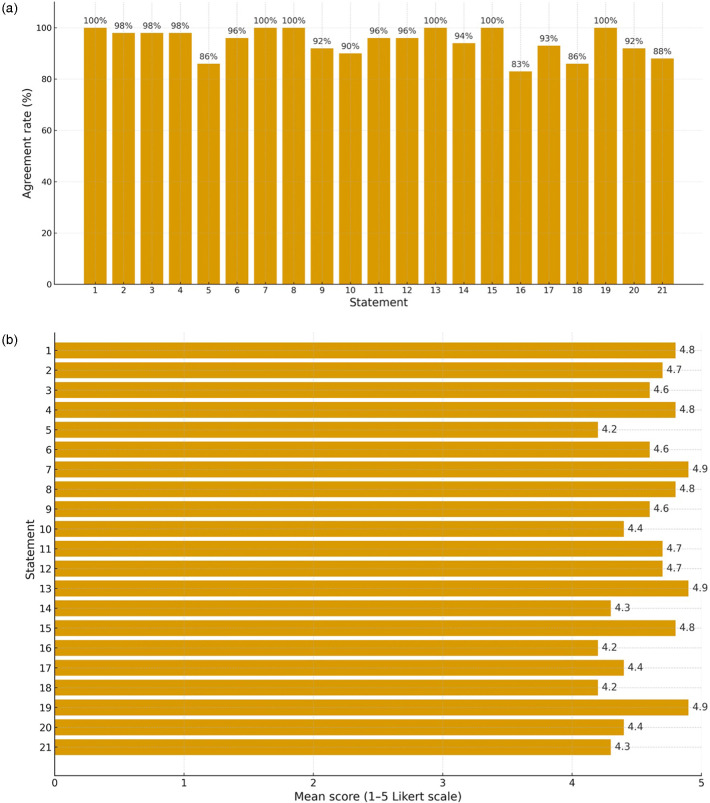
Distribution of agreement across the 21 statements included in the NoEmesi delphi consensus. The bar chart displays the percentage of panelists assigning a score of 4 or 5 (positive agreement) to each statement. All statements reached the predefined consensus threshold of ≥80% agreement, with individual values ranging from 83% to 100%. The highest levels of agreement were observed for statements related to the epidemiology and clinical relevance of acute gastroenteritis (AGE), metoclopramide pharmacology, and rapid symptom relief **(a)** and mean Likert scores for all statements included in the NoEmesi Delphi Consensus. The figure illustrates the mean agreement scores (range 1–5) for the 21 statements. Mean values ranged from 4.2 to 4.9, indicating uniformly high endorsement across the panel. Statements addressing pathophysiology, metoclopramide's mechanism of action and dosing, and the clinical burden of vomiting generated the highest mean scores **(b).**

**Figure 2:**
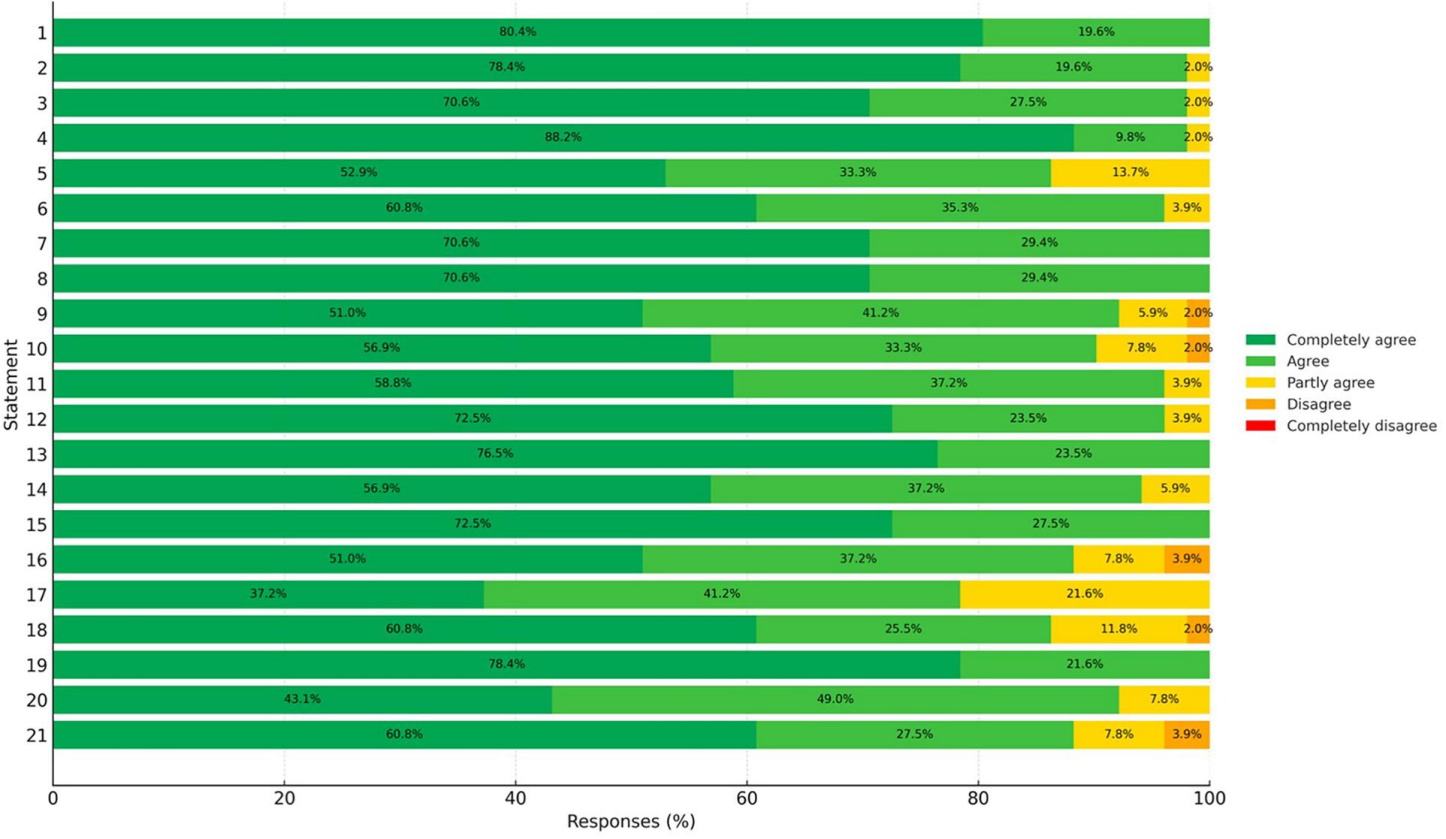
Likert-scale distribution of responses to the 21 statements of the NoEmesi delphi consensus. The figure illustrates the distribution of panel responses across the five-point Likert scale (from “completely disagree” to “completely agree”) for each of the 21 statements included in the NoEmesi Delphi Consensus. Bars represent the proportion of respondents selecting each response category, with darker shades of green indicating stronger agreement. Overall, agreement was consistently high across statements, with most responses falling within the “agree” or “completely agree” categories and only minimal proportions selecting “partly agree,” “disagree,” or “completely disagree.” This distribution reflects the strong consensus achieved across all statements and highlights the uniform endorsement of the clinical and pharmacological recommendations formulated by the expert panel.

All 70 invited panelists completed Round 2 voting (response rate: 100%). No missing responses were recorded for any of the 21 statements. Round 3 consisted of structured feedback and discussion without additional voting; therefore, no further response rate calculation was applicable. All 21 statements exceeded the predefined consensus threshold of 80% positive agreement. Levels of agreement ranged from 83% to 100%, with mean Likert scores between 4.2 and 4.9. No statements received a rating of 1 (strong disagreement). Detailed results for each statement are reported in Table 1, while the distribution of agreement across statements is visually summarized in Figures 1–2.

### 3.1 Clinical relevance and burden of vomiting in pediatric AGE

Statements addressing the epidemiology and clinical relevance of vomiting in pediatric AGE achieved the highest levels of agreement. Statement 1 reached 100% agreement (mean score 4.8), reflecting unanimous recognition of AGE as a common inflammatory condition characterized by gastrointestinal symptoms including vomiting, nausea, diarrhea, dehydration, and abdominal pain. Statements 2 and 3 achieved 98% agreement (mean scores 4.7 and 4.6, respectively), underscoring the high prevalence of vomiting, its impact on parental concern, and the associated socioeconomic burden related to dehydration and hospitalization.

Statement 4 also reached 98% agreement (mean score 4.8), confirming the panel's consensus that, although AGE is generally self-limiting and often manageable at home with ORT, persistent vomiting may necessitate escalation to intravenous rehydration. Statement 19, highlighting the importance of rapid symptom control to reduce child distress and parental anxiety, achieved 100% agreement with the highest mean score (4.9). Collectively, these findings demonstrate a strong and homogeneous consensus regarding the clinical burden of vomiting and its central role in management decisions.

### 3.2 Role of antiemetics in supporting home management

Statements addressing the potential role of antiemetic therapy in facilitating hydration and reducing emergency department referral showed robust, though slightly lower, agreement. Statements 5 and 18 each achieved 86% agreement, both with a mean score of 4.2. These results reflect a shared clinical perspective that pharmacological support may be appropriate in selected cases of persistent, uncontrollable vomiting that prevents effective ORT, provided that treatment is administered under medical supervision and within good clinical practice standards.

### 3.3 Pathophysiological rationale for antiemetic therapy

Consensus was also strong regarding the neurobiological mechanisms underlying nausea and vomiting. Statement 6 achieved 96% agreement (mean score 4.6), confirming widespread understanding of the central and peripheral neurochemical pathways involved and supporting the pharmacological rationale for serotonergic and dopaminergic antagonists in symptom control.

### 3.4 Metoclopramide: pharmacology, safety, and dosing

Statements addressing the pharmacological properties and clinical use of metoclopramide demonstrated consistently high levels of agreement. Statements 7 and 8 achieved 100% agreement (mean scores 4.9 and 4.8, respectively), reflecting unanimous recognition of metoclopramide's mechanism of action as a dopaminergic antagonist with additional serotonergic effects and gastroprokinetic activity.

Statement 9 reached 92% agreement (mean score 4.6), and Statement 10 achieved 90% agreement (mean score 4.4), confirming awareness of the drug's safety profile and the relevance of formulation changes, particularly the withdrawal of oral drops in favor of syrup formulations designed to reduce dosing errors. Statements 11 and 12 both reached 96% agreement (mean score 4.7), emphasizing the importance of age-related risk factors for adverse events and the critical role of caregiver education in early recognition of potential side effects.

The standard pediatric dosing regimen for metoclopramide was strongly endorsed. Statement 13 achieved 100% agreement (mean score 4.9), and Statement 14 reached 94% agreement (mean score 4.3), confirming broad acceptance of recommended dosing limits and formulations. Statement 20, addressing evidence supporting metoclopramide efficacy, including oral formulations, achieved 92% agreement (mean score 4.4). Statement 21, which considered the use of metoclopramide in children with AGE and persistent vomiting unresponsive to standard measures, reached 88% agreement (mean score 4.3), indicating cautious but positive consensus for its use strictly under medical supervision and with appropriate parental counseling.

### 3.5 Other antiemetic agents and non-pharmacological options

Regarding other antiemetics, Statement 15, addressing domperidone's restricted indications and its potential to prolong the QT interval, achieved complete consensus (100%) with a mean score of 4.8. Statement 16, concerning ondansetron's approved indications, potential risks, including QT prolongation, worsening of diarrhea, and rare serotonin syndrome, and limited pediatric data outside oncology settings, achieved 83% agreement with a mean score of 4.2. This represented the lowest level of consensus among all statements, although still above the predefined threshold, suggesting greater variability in clinical perceptions of ondansetron use outside its core labeled indications.

Statement 17, addressing ginger (standardized ginger extracts) as a possible non-pharmacological option for home management of vomiting despite limited supporting evidence, achieved 93% agreement with a mean score of 4.4.

### 3.6 Overall consensus patterns

Overall, agreement across the 21 statements was consistently high, ranging from 83% to 100%, with mean Likert scores between 4.2 and 4.9. The strongest consensus was observed for statements addressing epidemiology, clinical burden, and pathophysiological mechanisms of vomiting, whereas statements involving therapeutic nuances (particularly regarding ondansetron and the selective use of metoclopramide) showed slightly lower but still substantial levels of agreement.

## 4 Discussion

This multidisciplinary Delphi consensus underscores that vomiting in pediatric AGE, often preceded by nausea, is not merely an accompanying symptom but a key determinant of successful home management. By increasing child distress and caregiver anxiety and by directly undermining ORT, vomiting may accelerate dehydration risk and contribute to emergency department referral. Although current ESPGHAN guidance provides extensive recommendations for dehydration assessment (5) and ORT, practical direction on the home management of vomiting (28–31) and the role of antiemetics remains limited.

Importantly, the relative prominence of metoclopramide within the consensus statements reflects current Italian outpatient prescribing patterns and regulatory availability rather than an intended therapeutic hierarchy. In Italy, metoclopramide remains one of the few antiemetic agents accessible for home use in children under defined safety restrictions. However, this consensus should not be interpreted as implying superiority of metoclopramide over other antiemetic agents. Indeed, the strongest international evidence, including a Cochrane review, supports ondansetron for reducing vomiting and the need for intravenous rehydration, particularly in emergency department settings (14). The European Medicines Agency (EMA) introduced restrictions in 2013 limiting the pediatric use of metoclopramide due to the risk of neurological adverse reactions, particularly extrapyramidal symptoms (EPS). These restrictions include dose limitations, treatment duration not exceeding five days, and caution in younger children. The present consensus explicitly incorporates these regulatory constraints and emphasizes strict adherence to recommended dosing, short duration of therapy, and careful caregiver counseling. At the same time, we acknowledge that ondansetron's safety profile, particularly QT prolongation in the setting of electrolyte imbalances—requires careful consideration if its use were to be considered in outpatient settings.

Available evidence suggests that ondansetron has stronger support in the context of AGE (32, 33), particularly in emergency department settings, where randomized controlled trials and meta-analyses have demonstrated reductions in vomiting episodes, intravenous rehydration, and hospital admission. However, most of this evidence derives from supervised hospital environments rather than home management scenarios. The applicability of these findings to outpatient primary care settings remains less clearly defined.

With regard to safety, extrapyramidal adverse events associated with metoclopramide are generally uncommon and typically reversible upon drug discontinuation, but their potential severity justifies a cautious and individualized risk–benefit assessment (34, 35). The consensus therefore does not advocate routine antiemetic use, but rather a selective approach in cases of persistent vomiting that prevents effective oral rehydration.

International guidelines, including ESPGHAN recommendations, prioritize ORT as first-line therapy and provide limited endorsement of antiemetics outside specific clinical contexts (5). The present document aligns with this hierarchy, positioning pharmacological therapy as an adjunctive, case-dependent option rather than a replacement for established standards of care.

The NoEmesi Project was designed to address this gap by generating pragmatic, clinician-oriented statements that integrate symptom burden, pathophysiological rationale, and medication safety into a coherent approach for outpatient care. The uniformly high agreement observed across all statements, summarized in Figures 1–2, supports a shared clinical view that vomiting should be prioritized when it compromises rehydration and family coping. In this context, structured caregiver counseling emerges as a core component of management alongside ORT. Importantly, the consensus does not endorse a uniform pharmacological approach but rather supports individualized decision-making based on clinical context, regulatory frameworks, and available evidence, acknowledging that while ondansetron has the strongest international evidence in supervised settings, metoclopramide remains a commonly used option in Italian outpatient practice under strict safety precaution.

A central output of this consensus is a cautious, selection-based approach to antiemetic use. Panelists agreed that pharmacological treatment should not be routine in pediatric AGE, but may be considered in carefully selected cases of persistent, uncontrollable vomiting that prevents effective ORT, following individualized risk–benefit assessment and within regulatory and good clinical practice safeguards. This interpretation is consistent with the overall consensus pattern, with the strongest agreement on clinical burden and mechanistic rationale and slightly greater variability for statements involving therapeutic nuances.

Within this framework, metoclopramide was identified as a potential option in selected children, provided that its use is guided by strict dosing limits, age-related risk assessment, and active caregiver education to recognize early neurological adverse symptoms and promptly discontinue treatment if needed. Importantly, these statements position antiemetics as targeted support to facilitate rehydration and symptom relief under medical supervision, rather than as routine therapy. To ensure safe outpatient implementation, caregiver education must extend beyond general warnings to include a clear and actionable protocol for recognizing and managing potential adverse events, particularly EPS. Healthcare professionals should provide both verbal and written instructions covering the following key elements. First, caregivers should be instructed on the early warning signs of EPS, including restlessness, involuntary muscle spasms (affecting the neck, face, or eyes), stiff limbs, abnormal posturing, or any unusual movements. Second, it is important to explain the expected time course, that if EPS occur, they typically appear within 24–48 h of the first dose. Third, clear recommended response steps should be provided: if any of these signs appear, the caregiver must immediately discontinue the medication, reassure the child, and continue to encourage small, frequent sips of oral rehydration solution (ORS). Finally, and most critically, caregivers must be given explicit criteria for when to seek urgent medical attention. These include difficulty breathing, severe muscle rigidity preventing drinking, inability to walk, or any alteration in mental status. Caregivers should be strongly advised not to attempt home management of suspected extrapyramidal reactions and to seek urgent medical evaluation without delay. The provision of both clear written and verbal instructions is essential to ensure timely recognition and appropriate escalation of care, thereby maximizing the safety of any antiemetic use in the home setting.

The lower, albeit still above-threshold, consensus observed for ondansetron-related statements suggests ongoing variability in clinical perceptions of its role outside oncology and emergency settings, whereas the complete agreement on domperidone-related restrictions reflects broad awareness of age/weight limitations and QT-related safety concerns (36, 37). A critical safety consideration, is the synergistic risk between antiemetic-induced QT prolongation and the electrolyte disturbances (hypokalemia and hypomagnesemia) commonly resulting from fluid losses in pediatric AGE. As children with severe or prolonged diarrhea are inherently at an increased risk of cardiac arrhythmias due to these imbalances, the addition of an antiemetic with QT-prolonging potential, such as ondansetron (or domperidone) can significantly exacerbate this risk (37, 38). Therefore, this interaction represents a key consideration in therapeutic decision-making and reinforces the importance of carefully assessing the child's hydration and electrolyte status before prescribing such agents.

If complementary therapies such as ginger are considered, only standardized, pharmaceutical-grade preparations with defined concentrations of active constituents (gingerols and shogaols) should be used (39–41). The use of non-standardized, food-based products cannot be endorsed due to their unpredictable efficacy and safety profile. Unregulated herbal preparations may exhibit significant variability in the concentration of active constituents and carry a potential risk of contamination with environmental or toxic substances. Accordingly, clear guidance emphasizing the importance of product quality, specifically the use of standardized, pharmaceutical-grade extracts with defined concentrations of active compounds, is essential to minimize avoidable toxicological risks.

This study represents the first multidisciplinary Delphi consensus specifically focused on the home management of nausea and vomiting in pediatric AGE within a defined outpatient framework. Strengths include national representation across multiple pediatric subspecialties, a structured three-phase methodology, predefined consensus thresholds, and anonymous voting procedures designed to minimize dominance bias. The multidisciplinary participation and consistently high levels of agreement across clinically relevant domains provide a structured reference to support real-world outpatient decision-making.

However, several limitations inherent to the Delphi methodology should be acknowledged. First, the educational webinar conducted prior to voting, although designed to provide balanced and evidence-based information, may have influenced the direction of panel responses. While conflicting evidence and safety considerations were presented transparently, the standardization of pre-vote content introduces a potential framing effect that may shape interpretation and voting behavior. Second, panel composition may have influenced consensus outcomes. The substantial representation of primary care pediatricians reflects the outpatient focus of this initiative but may also orient perspectives toward community prescribing practices. Pediatric pharmacologists and emergency medicine specialists were less represented, potentially limiting the depth of pharmacokinetic and emergency care perspectives within the discussion. As with any Delphi process, the balance of specialties inevitably contributes to emphasis and interpretative framing. Third, the exclusively national (Italian) scope may limit external validity. Regulatory frameworks, drug availability, prescribing habits, and healthcare organization differ internationally; therefore, these findings may not be directly generalizable to healthcare systems with different regulatory environments or therapeutic accessibility. Fourth, the consensus reflects structured expert opinion rather than outcome-validated clinical effectiveness. No prospective patient-level validation or real-world outcome assessment was performed to confirm the clinical impact of the endorsed statements. Consensus cannot replace evidence derived from large randomized controlled trials specifically addressing home management of vomiting in pediatric AGE.

Finally, all statements exceeded the predefined 80% agreement threshold. While this indicates strong alignment among participants, it also raises the possibility of consensus inflation, particularly in areas where clinical practice patterns are well established. Statement formulation and the predefined agreement threshold may also influence the likelihood of achieving consensus. Overall, these findings should be interpreted as expert-informed guidance within a defined regulatory and outpatient context rather than as definitive comparative therapeutic recommendations or evidence of superiority among antiemetic agents.

In conclusion, this multidisciplinary Delphi consensus provides a structured and safety-oriented framework for the outpatient management of nausea and vomiting in children with AGE. ORT remains the cornerstone of care and the primary strategy to prevent dehydration (42). Antiemetic therapy should not be routine but may be considered in carefully selected cases of persistent vomiting that compromises effective rehydration, following individualized risk–benefit assessment and strict adherence to regulatory restrictions. Within the current Italian outpatient context, metoclopramide represents one potential option under defined dosing limits and short treatment duration, accompanied by explicit caregiver education and clear escalation protocols for adverse events. At the same time, available evidence supporting ondansetron, particularly in emergency department settings, should be acknowledged (43), and QT-related safety considerations must be integrated into clinical decision-making, especially in the presence of electrolyte disturbances.

These recommendations should be interpreted as expert-informed guidance rather than as evidence of therapeutic superiority. By integrating symptom burden, pharmacological rationale, regulatory constraints, and practical safety measures, this consensus aims to support clinicians and families in making cautious, context-appropriate decisions in the home management of pediatric AGE.

## Data Availability

The raw data supporting the conclusions of this article will be made available by the authors, without undue reservation.
